# Supplementary material for: Nationwide monthly burned area monitoring in Indonesia using Sentinel-2
Source: PLoS One. 2026 Apr 8;21(4):e0331831. doi: 10.1371/journal.pone.0331831 (PMC13061323; doi:10.1371/journal.pone.0331831)
Supplement: S1 Text — Description of the processing pipeline used to integrate the results into the Nusantara Atlas platform. (DOCX) [file pone.0331831.s001.docx]

# Supplementary Methods

# Processing Pipeline for Monthly Burned Area integration into Nusantara Atlas

This supplementary text describes the implementation of an **operational, largely automated processing system** for monthly burned-area mapping, integrating satellite imagery, active fire detections, cloud-based geospatial processing, and web-based dissemination through the Nusantara Atlas platform (<https://map.nusantara-atlas.org/> ).

While the analytical components (image compositing, classification, filtering, and validation) are described in detail in the main article, this section focuses on the **operational execution, automation, and system integration** of the processing chain.

The system is designed to operate on a **fixed monthly schedule**, with execution initiated automatically on the **5th day of each month**. This timing accounts for the typical latency in Sentinel-2 data availability, ensuring that imagery covering the full preceding month is included in the analysis.

The processing chain is executed using a set of **scripted workflows**, coordinated through scheduled Python scripts running on a cloud-based virtual machine (VM). Together, these scripts form an operational pipeline that manages data ingestion, processing, post-processing, and dissemination.

Routine operation does not require manual interaction. **Human involvement is limited to exception handling**, such as responding to system failures or execution errors. In such cases, the system generates automatic email notifications to alert operators.


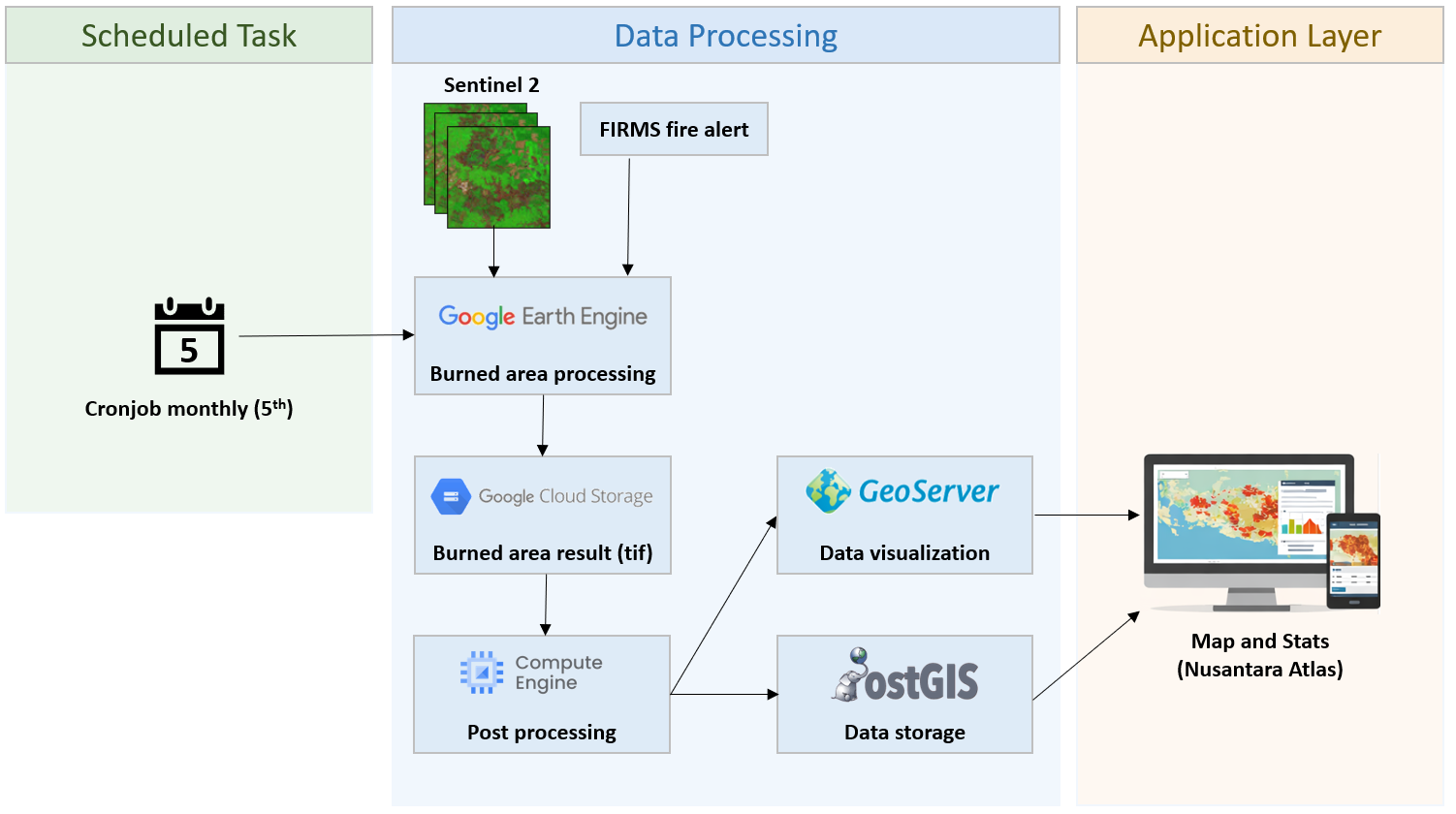
**Fig 1**. Diagram illustrating the overall system architecture and data flow.

This diagram presents a schematic of the overall system architecture and data flow. The architecture illustrates the interaction between external data sources (Sentinel-2 and FIRMS), the Google Earth Engine processing environment, cloud storage, post-processing services, and the Nusantara Atlas platform.

## Data Ingestion and Processing Environment

Two primary datasets are used in the burned area mapping process:

- Sentinel-2 surface reflectance imagery, accessed directly within the Google Earth Engine (GEE) data catalog.
- FIRMS active fire detections, obtained from the FIRMS database and ingested into Google Earth Engine (GEE) as an asset.

Rather than relying on direct downloads from the FIRMS server, which are limited to short temporal windows, FIRMS data are retrieved automatically on a continuous daily basis using a scheduled script and stored in a local database. This approach enables flexible querying over extended time ranges and supports dynamic integration of FIRMS data into GEE for subsequent processing.

All image compositing, Random Forest classification, and burned-area extraction are performed within Google Earth Engine, which serves as the core analytical and computational environment. Burned-area outputs are intersected with land-cover datasets to characterize the affected land types.

The outputs of the GEE processing stage are raster datasets in GeoTIFF format, which are automatically exported to **Google Cloud Storage (GCS)**, where they are accessed by a Google Compute Engine virtual machine for subsequent processing.

## Post-Processing

Following export of the result in GeoTIFF format, automated Python scripts running on a Google Compute Engine virtual machine perform two main tasks :

1. Statistical aggregation, including calculation of burned area metrics by administrative boundaries, concessions, protected areas and other spatial units. The resulting statistics are written directly on a database.
2. Generation of image pyramids, optimized for efficient visualization in web mapping applications.

Operational monitoring is handled through automated execution checks. The system verifies the successful completion of each processing step and relies on failure detection and email notifications to flag issues requiring human attention.

## Integration with the Nusantara Atlas Platform

The burned-area products are disseminated through the **Nusantara Atlas** web platform, which is programmatically connected to the processing outputs. Raster datasets are accessed directly from Google Cloud Storage, while statistical summaries are retrieved from the database populated during post-processing.

Once the processing pipeline completes successfully, updated burned-area layers and statistics become available on the Nusantara Atlas website **without manual data transfer steps**, enabling timely public access to monthly burned-area information.
