# Supplementary material for: Nationwide monthly burned area monitoring in Indonesia using Sentinel-2
Source: PLoS One. 2026 Apr 8;21(4):e0331831. doi: 10.1371/journal.pone.0331831 (PMC13061323; doi:10.1371/journal.pone.0331831)
Supplement: Attachment — Submitted filename: Response to Reviewers.docx [file pone.0331831.s002.docx]

**Response to Reviewers**

**Manuscript title:** *Automated monthly burned area mapping for Indonesia using Sentinel-2 and FIRMS data (2019–2024)*
**Manuscript ID:** PONE-S-25-59671
**Authors:** D.L.A. Gaveau, A. Descals, M.A. Salim
**Journal:** *PLOS ONE*

**General Response**

We thank the Academic Editor and both reviewers for their constructive and insightful comments. We have revised the manuscript accordingly. Below we address each comment point-by-point. Reviewer comments are reproduced in *italics*, followed by our responses in plain text.

**Reviewer #1**

***“In remote sensing and computer science, algorithm often implies a novel mathematical or computational procedure. In this manuscript, the approach is essentially a workflow combining preprocessing, machine learning, and rule-burned-area filtering… The claim of developing a new ‘algorithm’ is overstated.”***

**Response:**
We appreciate this clarification. We agree that the term *workflow* or *processing chain* more accurately describes our method. We have revised the manuscript throughout to reduce the emphasis on “new algorithm” and instead describe it as an “automated monthly burned-area processing chain” built from established methods — Sentinel-2 pre/post-fire compositing, Random Forest classification, and rule-burned-area filtering — integrated into a single operational workflow.
The novelty lies in (i) its automation at national scale, (ii) its monthly temporal resolution, and (iii) its adaptation to Indonesia’s landscape and policy requirements.

***“If the authors claim this is the first automated nationwide monthly burned area algorithm, readers will expect at least a description of how end users can interact with it… Without an interface or demonstration, the system risks being perceived as a research prototype.”***

**Response:**
We agree and have clarified in the revised manuscript how the system will be made accessible to end users. The automated workflow will being integrated into the **Nusantara Atlas** platform ([www.nusantara-atlas.org](http://www.nusantara-atlas.org)), which already provides near-real-time deforestation and fire-alert data. We will publish the **monthly burned-area layers** on Nusantara Atlas. This will ensure operational accessibility for the public, NGOs and relevant agencies. To reflect this, the Abstract now includes:

“*Monthly burn-scar updates (post-December 2024 to present) are available for viewing and analysis via Nusantara Atlas (*[*www.nusantara-atlas.org*](http://www.nusantara-atlas.org)*), where the system operates as an open, operational platform for public use..*”

The Discussion also now concludes with a paragraph explaining the implementation and access considerations in full detail: *“To support wider adoption, we have integrated our burned-area maps into the Nusantara Atlas platform (*[*www.nusantara-atlas.org*](http://www.nusantara-atlas.org)*), which already provides near-real-time deforestation and fire hotspots data. This integration will enable continuous updates of monthly burned-area maps and make them accessible for analysis by government agencies, NGOs, and the public.*

***“The link between ENSO (ONI) and IOD anomalies and fire peaks is described as correlation, but no statistical modeling is provided to quantify strength of influence.”***

**Response:**
We thank the reviewer for this comment. We compared the ENSO (ONI) and IOD anomalies with the burned area monthly as an additional validation exercise to verify that the burned area seasonality aligns with the expected fire season patterns linked with ENSO and IOD and reported in previous studies. Our goal was not to model the statistical strength of influence but to confirm that the temporal variations in burned area are consistent with known large-scale climate drivers. We have clarified this point in the Discussion section. The revised text now explicitly notes that the analysis is descriptive.

We wrote: *“We note that this analysis is descriptive and intended to illustrate temporal co-variation between climatic indices and monthly burned-area fluctuations, rather than to quantify causality. Future work will apply statistical modelling approaches incorporating additional climatic variables such as air temperature, rainfall, vapor pressure deficit, or climatic water deficit, known drivers of fire occurrence,to estimate the strength and causality of these climatic influences.”*

**Reviewer #2**

***“The abstract does not mention the methodology.”***

**Response:**
We have revised the abstract to include a concise description of the method used to map burned areas:

*“**Our approach uses a Random Forest model that classifies Sentinel-2 imagery and uses FIRMS fire hotspots to reduce false positives. The resulting 20-m monthly burned-area maps cover the entire country from January 2019 to December 2024.”*

***“Check the text for language issues.”***

**Response:**
The manuscript has been carefully read to correct minor grammatical and typographical issues, ensuring clear and standard English throughout.

***“Line 67: Provide some references regarding the use of Landsat data in Indonesia.”***

**Response:**
We have added the following reference to support the statement about Landsat’s use for burned-area mapping in Indonesia by the Indonesian government:

https://sipongi.gakkum.kehutanan.go.id/indikasi-luas-kebakaran

In this reference, it is written: “The area of forest and land fires is calculated burned-areas on Landsat-8 OLI/TIRS image analysis”

***“Line 81: ‘with advanced machine learning algorithms’ – State exactly which methods were used.”***

**Response:**
We have clarified that the workflow uses the Random Forest classifier implemented in Google Earth Engine. The sentence now reads: “a method that combines time-series Sentinel-2 imagery with a machine learning classification model (Random Forest), implemented within the Google Earth Engine.”

***“Line 91: ‘To date, no automated burned area detection system exists for Indonesia.’ I found several papers using AI for burnt area detection… How can the authors claim that their method is the only one?”***

**Response:**
We appreciate this correction; and have revised the sentence for accuracy.

We now state: “*While previous studies have proposed promising methodologies for monthly burned-area mapping in Indonesia, using Sentinel-2 and other remote sensing inputs (Vetrita et al., 2025; Arjasakusuma et al., 2022), none have yet delivered an automated, nationwide, monthly burned-area mapping system that operates continuously.*”

This distinguishes our contribution in terms of spatial coverage, temporal frequency, and operational automation.

***“Line 94: Explain the ‘FIRMS’ dataset, as it might be new to some readers.”***

**Response:**
We have added the following text that explains the FIRMS dataset:

We now say in the Introduction: “*Our method integrates high-frequency Sentinel-2 time series (every 2–5 days) with FIRMS (Fire Information for Resource Management System) daily fire alerts (Giglio et al., 2016), which are derived from MODIS (Moderate Resolution Imaging Spectroradiometer) sensors onboard NASA’s Terra and Aqua satellites and the VIIRS (Visible Infrared Imaging Radiometer Suite) sensors onboard the Suomi NPP and NOAA-20 satellites (NASA, 2025). These instruments detect daily thermal infrared anomalies associated with active combustion. In this study, the resulting fire hotspot detections serve as both spatial and temporal filters for identifying candidate burned areas detected and delineated using Sentinel-2 imagery.*”

***“What is the difference between this paper and the previous one (line 79)?”***

**Response:**
We now clarify this distinction in the Introduction section:

“Our earlier study (Gaveau et al., 2021) produced an **annual** burned-area product suitable for retrospective analysis, whereas the present study develops a **monthly, automated system** designed for near-real-time monitoring, enabling continuous national updates.”

***“Figure 1 is very simple. The paper is fine without it as it is. Consider adding more detail or removing it.”***

**Response:**
We have removed Figure 1.

***“The text uses many abbreviations without explaining them fully.”***

**Response:**
We have ensured that all abbreviations (e.g., GEE, FIRMS, NBR, ONI, IOD, etc.) are defined upon first use in the manuscript.

We replace the BA abbreviation with “burned-area”

***“I don't understand why 6.25 hectares was selected as the threshold…”***

**Response:**
We thank the reviewer for raising this point.

Our classification system can detect burn scars smaller than 6.25 ha; however, we adopted this threshold to align with the official minimum mapping unit (MMU) used by the Indonesian Ministry of Forestry for national burned-area assessments.
This ensures comparability with official datasets and legal frameworks. Smaller patches are filtered from the published layer to meet this national standard.

The revised manuscript now makes this clear in the Introduction section: “ *The workflow generates monthly burn-scar maps at 20-meter resolution, with a minimum mapping unit of 6.25 hectares — aligned with the official minimum burn-scar size recognized by Indonesian authorities*”

(MoEF): Indonesia’s Second Forest Reference Emission Level (FREL) Submission to the UNFCCC, Ministry of Environment and Forestry (MoEF), Jakarta, Indonesia, 2022. [https://redd.unfccc.int/files/modified_2nd_frl_indonesia_20220529_clean.pdf](https://redd.unfccc.int/files/modified_2nd_frl_indonesia_20220529_clean.pdf?utm_source=chatgpt.com)

***“In section 3.2, please explain what happens in the different phases of each oscillation regarding precipitation, temperature, etc., and how these factors affect burned areas.”***

**Response:**
We have added a paragraph in Section 3.2 explaining why positive IOD and El Niño (ONI) phases typically increase fire risk, while negative phases reduce it.

We wrote in Section 3.2: *“During positive IOD and El Niño phases, reduced convection and rainfall lead to drier and warmer conditions across much of Indonesia, increasing fuel flammability and extending the dry season. Conversely, La Niña and negative IOD phases enhance rainfall and humidity, reducing fire occurrence.”*

This provides the climatic context for the observed burned-area variations. We note in the Discussion section that future work will apply statistical modelling approaches incorporating additional climatic variables such as air temperature, rainfall, vapor pressure deficit, or climatic water deficit, known drivers of fire occurrence (Descals et al., 2022), to estimate the causality of these climatic influences.

**Data Availability**

Reviewer #1 expressed concern that data were not fully available.
*We have clarified in the* ***Data Availability Statement*** *that:*

- All input datasets (Sentinel-2 SR, FIRMS, MODIS MCD64A1) are **publicly accessible**.
- The monthly burned-area outputs will be hosted openly via the Nusantara Atlas upon acceptance. Thus, we will include the link to the dataset in the accepted version.

We believe these revisions address all reviewer concerns and substantially improve the manuscript’s clarity, rigor, and accessibility.
