# Supplementary material for: Nationwide monthly burned area monitoring in Indonesia using Sentinel-2
Source: PLoS One. 2026 Apr 8;21(4):e0331831. doi: 10.1371/journal.pone.0331831 (PMC13061323; doi:10.1371/journal.pone.0331831)
Supplement: Attachment — Submitted filename: Second Response to Reviewers.docx [file pone.0331831.s003.docx]

**Response to Reviewers**

**Manuscript title:** Nationwide Monthly Burned Area Monitoring in Indonesia Using Sentinel-2
**Manuscript ID:** PONE-D-25-45724R1
**Authors:** D.L.A. Gaveau, A. Descals, M.A. Salim
**Journal:** *PLOS ONE*

**General Response**

We thank the Academic Editor and both reviewers for their constructive and insightful comments. We have revised the manuscript accordingly. Below we address each comment point-by-point. Reviewer comments are reproduced in *italics*, followed by our responses in plain text.

**Reviewer #1**

***“The authors provide a detailed narrative of analytical steps… however these are described as analytical procedures rather than components of an automated processing system.”***

**Response:**
We thank the reviewer for this insightful comment. We agree that the original manuscript did not sufficiently distinguish between the analytical methodology and the operational implementation of the processing chain. In response, we have revised the manuscript and added a dedicated description of the operational system as Supplementary Information.

We clarified the operational and automated nature of the processing chain. We added Supplementary Methods, describing execution, scheduling, orchestration, and system integration. We added Figure S1, illustrating the system architecture and data flow. We revised terminology throughout (e.g., “operational”, “largely automated”) for precision

***“The manuscript does not clearly describe whether the processing chain runs automatically on a fixed schedule.”***

**Response:**
We have now explicitly described the execution schedule in Supplementary Methods S1. The system operates on a fixed monthly schedule, automatically initiated on the 5th day of each month, allowing for Sentinel-2 data latency.

***“How data ingestion, processing, quality control, and output publishing are orchestrated.”***

**Response:**
Supplementary Methods S1 now describes the orchestration of:

- Automated Sentinel-2 data access within Google Earth Engine
- Automated FIRMS hotspot retrieval and ingestion
- Scripted GEE processing workflows
- Automated post-processing via Python scripts
- Dissemination through Nusantara Atlas

***“*** ***Whether human intervention is required at any stage.”***

**Response:**
We have clarified that the workflow is largely automated, with human involvement limited to exception handling, such as system failures. No manual intervention is required during routine monthly execution.

***“No workflow diagram or system architecture figure is presented”***

**Response:**
We have added Figure S1, which presents a schematic of:

Data sources (Sentinel-2, FIRMS)

Google Earth Engine processing

Cloud storage

Post-processing

Nusantara Atlas dissemination

***“ The manuscript suggests that burned-area results are “made available” through the Nusantara Atlas website, but it remains unclear whether the website is dynamically connected… or manually uploaded.”***

Supplementary Methods S1 now clarifies that burned-area outputs are:

Exported automatically from GEE to Google Cloud Storage

Accessed programmatically by Nusantara Atlas services

We revised wording in the manuscript to state that products are made available via Nusantara Atlas, avoiding any implication of manual upload steps.

***“The authors rely on several independent systems (Google Earth Engine, FIRMS, and a web visualization platform), yet the manuscript does not explain how components are programmatically integrated”***

Supplementary Methods S1 now describes the integration pipeline, including:

Python-based scheduling and orchestration

Automated data exchange between GEE, cloud storage, and post-processing services

Programmatic linkage to Nusantara Atlas

We also clarify that Google Earth Engine serves as the core execution environment, with external scripts coordinating post-processing and dissemination.

***“Explicitly describe the nature of the processing system***

**Response:**

We have followed this recommendation by:

Separating analytical methodology (main manuscript)

Operational automation & architecture (Supplementary Methods)

Providing Figure S1 (workflow/system diagram)

We also revised terminology throughout the manuscript for clarity and accuracy.
